# Nedaplatin/*Peganum harmala* Alkaloids Co-Loaded Electrospun, Implantable Nanofibers: A Chemopreventive Nano-Delivery System for Treating and Preventing Breast Cancer Recurrence after Tumorectomy

**DOI:** 10.3390/pharmaceutics15102367

**Published:** 2023-09-22

**Authors:** Nada K. Sedky, Kholoud K. Arafa, Manal M. M. Abdelhady, Marwa Y. Issa, Nour M. Abdel-Kader, Noha Khalil Mahdy, Fatma A. Mokhtar, Mohammad Y. Alfaifi, Sherif Ashraf Fahmy

**Affiliations:** 1 Department of Biochemistry, School of Life and Medical Sciences, University of Hertfordshire Hosted by Global Academic Foundation, R5 New Garden City, New Administrative Capital, Cairo 11835, Egypt; 2 Drug Design and Discovery Lab, Zewail City for Science, Technology and Innovation, Cairo 12578, Egypt; 3 Clinical Pharmacy Department, Faculty of Pharmacy, Badr University, Cairo 11829, Egypt; 4 Department of Pharmacognosy, Faculty of Pharmacy, Cairo University, Kasr El-Aini Street, Cairo 11562, Egypt; 5 Department of Biochemistry, Faculty of Science, Ain Shams University, Cairo 11566, Egypt; 6 Department of Pharmaceutics and Industrial Pharmacy, Faculty of Pharmacy, Cairo University, Kasr El-Aini Street, Cairo 11562, Egypt; 7 Department of Pharmacognosy, Faculty of Pharmacy, El Saleheya El Gadida University, El Saleheya El Gadida 44813, Egypt; 8 Biology Department, Faculty of Science, King Khalid University, Abha 9004, Saudi Arabia; 9 Department of Chemistry, School of Life and Medical Sciences, University of Hertfordshire Hosted by Global Academic Foundation, R5 New Garden City, New Capital, Cairo 11835, Egypt

**Keywords:** nedaplatin (N), *Peganum harmala* alkaloids (L), nanofibers, breast cancer, apoptosis, tumerectomy

## Abstract

Currently, the main pillars in treating breast cancer involve tumorectomy pursued by hormonal, radio, or chemotherapies. Nonetheless, these approaches exhibit severe adverse effects and might suffer from tumor recurrence. Therefore, there is a considerable demand to fabricate an innovative controlled-release nano-delivery system to be implanted after tumor surgical removal to guard against cancer recurrence. In addition, combining platinum-based drugs with phytochemicals is a promising approach to improving the anticancer activity of the chemotherapeutics against tumor cells while minimizing their systemic effects. This study designed polycaprolactone (PCL)-based electrospun nanofiber mats encapsulating nedaplatin (N) and *Peganum harmala* alkaloid-rich fraction (L). In addition to physicochemical characterization, including average diameters, morphological features, degradation study, thermal stability, and release kinetics study, the formulated nanofibers were assessed in terms of cytotoxicity, where they demonstrated potentiated effects and higher selectivity towards breast cancer cells. The dual-loaded nanofiber mats (N + L@PCL) demonstrated the highest antiproliferative effects against MCF-7 cells with a recorded IC50 of 3.21 µg/mL, as well as the topmost achieved selectivity index (20.45) towards cancer cells amongst all the tested agents (N, L, N@PCL, and L@PCL). This indicates that the dual-loaded nanofiber excelled at conserving the normal breast epithelial cells (MCF-10A). The combined therapy, N + L@PCL treatment, resulted in a significantly higher percent cell population in the late apoptosis and necrosis quartiles as compared to all other treatment groups (*p*-value of ≤0.001). Moreover, this study of cell cycle kinetics revealed potentiated effects of the dual-loaded nanofiber (N + L@PCL) at trapping more than 90% of cells in the sub-G1 phase and reducing the number of cells undergoing DNA synthesis in the S-phase by 15-fold as compared to nontreated cells; hence, causing cessation of the cell cycle and confirming the apoptosis assay results. As such, our findings suggest the potential use of the designed nanofiber mats as perfect implants to prevent tumor recurrence after tumorectomy.

## 1. Introduction

Breast cancer is the most common type of cancer among women. Reliance on surgical resection, radiotherapy, and chemotherapy constitutes the pillars of conventional treatment protocols. Despite their clinical effectiveness, each of those procedures holds several inherent drawbacks. Chemotherapy serves as the foremost protocol for the effective treatment of cancer. Chemotherapeutic agents are mainly a class of low molecular weight xenobiotics with narrow therapeutic index, limited selectivity as well as short half-life time, which renders them highly toxic when applied systemically. To avoid such drawbacks, breast-conserving surgery (BCS) stands as the approach of preference to eradicate localized or early-stage breast cancers. Yet, local-regional recurrence is most likely to occur post-surgery, with a frequency of 10–20% of the cases within 5 years, reaching the daunting frequency of 50% within 10 years [1]. Radiation therapy has been adopted to prevent such a high recurrence rate of breast cancer. However, the hazardous side effects of radiation therapy adversely affect patient’s post-treatment health and impose a major concern advocating against its frequent clinical application [2]. 

Recently, it has been observed that utilizing nanofiber scaffolds loaded with anticancer drugs can be a gateway to reducing the probability of disease recurrence [3]. To obtain optimal effect, designing a system capable of locally releasing the drug over a relatively long period should be ensured. In this way, polymeric nanofibers (NFs) have been substantially employed as drug-loaded implantable patches for local cancer therapy [4,5]. Implantable NFs are considered advantageous as their preparation is cost-effective, their structure is highly porous, allowing for diffusivity of cargo as well as mechanical malleability, and they also possess a high surface-to-volume ratio which favors good drug-encapsulation [5,6]. All of these positive attributes have been previously reported for Poly-caprolactone (PCL) based NFs. PCL is a synthetic, hydrophobic, semi-crystalline, aliphatic polyester polymer that is used in biomedical applications and pharmaceuticals [7,8,9]. Compared to other polymers commonly used for nanofibers fabrication, including PEG and PVP, PCL is of higher hydrophobicity, alleviating the drawbacks of drug burst release [10]. Moreover, PCL exhibits good biocompatibility, biodegradability into safe metabolites, and unique mechanical properties, nominating it as a promising candidate for use in sub-dermal implants as well as great potential for breast reconstruction after partial mastectomy [11,12]. Additionally, PCL exhibits high similarity to the physiological extracellular matrix, making it a preferable drug carrier for localized chemotherapeutics delivery, fracture internal fixation devices, and tissue regeneration scaffolds [13,14]. 

**Scheme 1:**
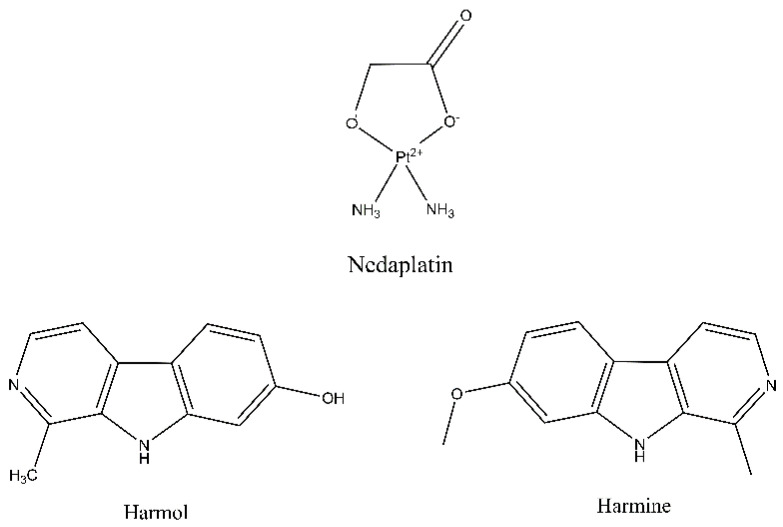
Chemical structures of nedaplatin, harmol, and harmine.

Nedaplatin (N) is a second-generation platinum-based antineoplastic drug capable of DNA cross-linking, thus hindering proper DNA synthesis and repair (Scheme 1) [15]. N is considered superior to other class members as it overcomes cancer drug resistance, possesses improved pharmacokinetics as well as pharmacodynamics, and exhibits lower side effects [16]. A clinical study by Cai et al. showed that the patients tolerated N-based chemotherapy well, as evidenced by the prolonged time-to-treatment failure, enhanced overall survival, and marginal improvement in the overall response rate [17]. Moreover, a recent review by Ivanova et al. mentioned that N plays a synergistic effect when administered in drug combination or along with radiotherapy and that it promotes an improved antitumor activity in the resected gynecologic carcinoma relative to cisplatin [18]. Moreover, compared to cisplatin, N was found to possess lower leukopenia, renal, neuronal, and gastrointestinal toxicities, which nominates it as a better alternative therapeutic agent [18]. It has been reported that N, combined with docetaxel, is among the most effective combination therapies to eradicate triple-negative breast cancer [19].

Unlike monotherapy of cancer, combination therapy has been employed to guard against developing resistant cancer cells and suffering severe adverse effects [18]. Since the plant-derived natural products were reported to enhance the ultimate outcome of cancer therapy, we sought to co-encapsulate *Peganum harmala* plant extract (L) with the chemotherapeutic drug (N) within the NF nanofiber. ß-carboline alkaloids, namely harmine and harmol, constitute the main phytochemical class of *P. harmala* (Scheme 1). 

ß-carboline alkaloids exhibit tumor antiproliferative activity through DNA intercalation, inhibition of the DNA repair topoisomerase enzymes, as well as apoptosis induction [20,21,22,23,24]. As demonstrated in the work of Vannozzi et al., harmala’s plant extract effectively halts the growth of triple-negative breast cancer through the induction of apoptosis [25]. Of more relevance to the tested cell line (MCF-7), it was previously reported that harmine, one of the main bioactive ß-carbolines, resulted in up-regulating p53 gene expression and apoptosis induction [26].

The novelty of our system lies in the attempt to benefit from the privileges of using combination therapy with the aim of evaluating the potential synergistic therapeutic effects that would result from such a mixture in treating breast cancer. Additionally, we have deployed the FDA-approved biodegradable polymer, PCL, to prepare a sustained-release implantable patch. To this extent, we report the synthesis of electrospun PCL-to-based drug-loaded nanofibers either individually or in combination. Comprehensive chemical characterization, mechanical stability testing, and antiproliferative activity evaluation against the MCF-7 cell line are to be investigated.

## 2. Materials and Methods

### 2.1. Materials

Polycaprolactone (average MW of 80,000) and nedaplatin were purchased from Sigma–Aldrich (St. Louis, MO, USA). Glacial acetic acid was provided by Merck Company (Darmstadt, Germany). Phosphate-buffer saline (PBS, pH 7.4) and acetate buffer (pH 5.4) were purchased from Lonza Bioscience (Walkersville, MD, USA). Dulbecco’s Modified Eagle Medium (DMEM) and fetal bovine serum (FBS) were purchased from Gibco (Thermoscientific, Steingrund, Dreieich, Germany). The penicillin–streptomycin mixture was obtained from Lonza Bioscience, Basel, Switzerland). The remaining reagents were all of analytical grade. Annexin V-FITC apoptosis detection kit was purchased from Abcam Inc. (Cambridge Science Park, Cambridge, UK). Propidium iodide was obtained from Calbiochem (Darmstadt, Germany).

### 2.2. Preparation of Peganum harmala Alkaloid-Rich Fraction (L)

Dried mature seeds of *P. harmala* L. were provided from the local Egyptian market. The alkaloid-rich fraction of *P. harmala* seeds was extracted as described previously. Briefly, *P. harmala* ground seeds (2.4 kg) were soaked in 70% ethanol three successive times until exhaustion. The extracts were collected, filtered, and evaporated using rotavap to yield 520 g residue. Part of this residue was subjected to acid/base extraction to yield a rich fraction of harmala alkaloids (L). Then, the extracted L fraction was characterized using ultraperformance liquid chromatography–electrospray ionization–tandem mass spectrometry (UPLC/ESI-MS) following our formerly reported protocol [24,27].

### 2.3. Preparation of Nanofiber Mats

#### 2.3.1. Preparation of Electrospinning Solutions

Polycaprolactone (PCL) solution (15 *w*/*v*%) was prepared by dissolving PCL in 90% acetic acid under magnetic stirring overnight at room temperature. For the drug/s-loaded PCL solutions, nedaplatin (N), harmala alkaloids (L), and N + L were dissolved slowly into the PCL solution (in a ratio of 2:15 drug to polymer weight ratio), forming N@PCL, L@PCL, and N + L@PCL, respectively. The prepared solutions were subjected to magnetic stirring overnight to form homogenous mixtures. 

#### 2.3.2. Electrospinning Process

**Scheme 2:**
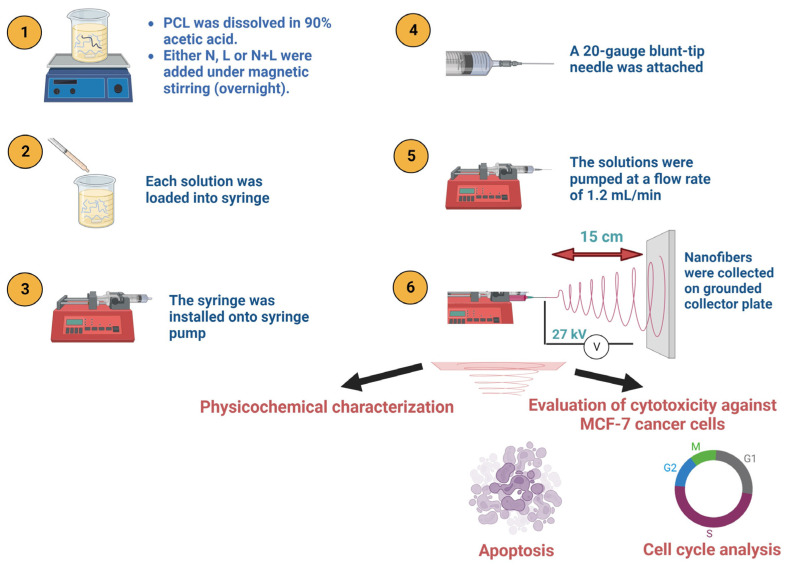
Schematic diagram demonstrating the steps involved in the design and characterization of different PCL nanofibers.

Electrospinning was conducted at 25 °C utilizing Nanon Electrospinner-01B (MECC Nanofiber, Fukuoka, Japan). Each PCL solution was loaded separately into a plastic syringe equipped with a 20-gauge blunt-tip needle. The solutions were fed using a flow rate of 1.2 mL/h. A high voltage supply provided a 27 kV voltage to the blunt-tip needle located 15 cm from the grounded collector plate. The fabricated nanofibers were stored at 4 °C for further investigation (Scheme 2).

### 2.4. Characterization and Loading Efficiency

The morphological features and average diameters of the prepared nanofiber mats were studied using scanning electron microscope (SEM), Quanta FEG250-FEI-USA, at an accelerating voltage of 20 kV. Coating of the nanofibers’ surfaces was performed using gold for imaging. 

The chemical features of the prepared nanofibers were evaluated using an FT-IR spectrophotometer (vertex 70 RAM II, Bruker Spectrometer) in the spectral range of 4000–500 cm^−1^. The thermal stability of the nanofibers was assessed utilizing a Q50 TGA Thermogravimetric Analyzer, USA. The fiber mats were located in a pan made of platinum and heated from 0 to 500 °C under a nitrogen atmosphere and a heating rate of 10 °C/min. The thermal events interpretation and the first derivative thermogravimetric (DTG) analysis of the weight loss as a function of temperature were performed by the OriginPro 8.5 software (OriginLab Corporation, Northampton, MA, USA), resulting in a thermogravimetric analysis–differential thermal analysis [TGA-DTA] curve. The first derivative peak temperatures (the inflection point) indicate the points of the most significant rate of change on the weight loss curve.

To determine the amounts of N and L in the nanofibers, nanofiber mats of 2 × 5 cm were first dissolved in 90% acetic acid. Then, the absorbance of N and L was measured at 220 and 368 nm, respectively (Figure S1), using UV–Vis spectrophotometer (Cary series UV-Vis-NIR, Agilent Technologies, Inc., Santa Clara, CA, USA). Finally, Equation (1) was utilized to determine the *drug loading* percentages.

(1) $$Drug\;Loading\;\left(\%\right)=\frac{Drug\;amount\;in\;each\;nanofiber}{Initial\;total\;drug\;amount\;in\;each\;nanofiber}$$

### 2.5. In Vitro Degradation Study

The in vitro degradation assay was carried out, as detailed previously, with some modifications [28,29]. In brief, 20 mg of each nanofiber was placed in a Falcon tub containing 5 mL 0.0067 M phosphate buffer saline (PBS, pH 7.4). The beaker was incubated at 37 °C and subjected to constant shaking at 125 rpm for 21 days. Fibers were withdrawn from the buffer medium at specific intervals, rinsed thrice with deionized water, and dried at 37 °C for 72 h. The weight loss % of the electrospun nanofibers was determined using Equation (2).

(2) $$Weight\;Loss\left(\%\right)=\frac{Initial\;Weight\;before\;incubation-Final\;weight\;after\;incubation}{Initial\;Weight\;before\;incubation}$$

### 2.6. In Vitro Release Study

The N and L release rates from the designed nanofiber mats were evaluated at two pH values (pH 7.4 and pH 5.4), as previously reported, with some modifications [30]. An equal amount of each nanofiber (20 mg) was placed individually in Falcon tubes containing 3 mL of the releasing medium (either 0.0067 M PBS or acetate buffer). Tween 80 was added to the releasing medium as an emulsifier that improves hydrophobic drug solubility and minimizes release resistance. Afterward, the Falcon tubes were placed in a shaking incubator (Jeio tech SI-300, Seoul, Republic of Korea) at 37.5 ± 0.5 °C and under a constant shaking of 120 rpm. 

At specific times, an amount of the release medium was retrieved, and the drugs (N and L) were quantified at 220 and 368 nm, respectively, using UV–Vis spectrophotometer (Cary series UV-Vis-NIR, Sydney, Australia). The releasing medium was replaced with fresh buffer every 24 h to keep sink conditions, and this study was conducted for a period of 10 days. 

### 2.7. Release Kinetics

The percentages of N and L values acquired from the in vitro release study were further evaluated utilizing five mathematical kinetic models to examine N and L release patterns from dual-loaded PCL nanofibers. In this context, zero-order, first-order, Higuchi model, Korsmeyer–Peppas, and Hixson–Crowell kinetic models were used, as detailed elsewhere, adopting Equations (3)–(7) [31,32].

(3) $$C=K_{o}\;t$$

(4) Log 100−C=−Kf t 2.303

(5) $$C=K_{H}\sqrt{t}$$

(6) $$C=K_{k}\;t^{n}$$

(7) $$\sqrt[{3}]{W_{0}}-\sqrt[{3}]{W_{t}}=K_{ß}\;t$$

where C is the cumulative % drug released at time t; K_0_ is the zero-order rate constant; K_f_ is the first-order rate constant; K_H_ is the Higuchi constant; K_K_ is the Korsmeyer–Peppas constant; n is the exponent that describes a particular diffusion mechanism; W_0_ is the initial amount of drug in the system; W_t_ is the remaining amount of drug in the system at time t; and K_ß_ is the Hixson-Crowell release constant. 

### 2.8. Cell Culture

Breast cancer cell line (MCF-7), as well as normal breast epithelial cell lines (MCF-10A), were maintained in RPMI 1640 complete culture medium. Such medium contained FBS at a concentration of 10% *v*/*v* together with penicillin–streptomycin at 0.01% *w*/*v* concentration. All were kept in a 5% CO_2_ incubator at 37 °C.

### 2.9. Cytotoxicity Assay 

Cell viability of drug-loaded nanofibers and free drug suspension was tested using SRB assay on MCF-7 and MCF10A cell lines 72 h after treatment. Nanofibers were exposed to UV light for sterilization. MCF-7 and MCF10A cells (5 × 10^3^ cells/well) were added to the RPMI culture medium in the 96-well plates. The plates were left in the incubator at 37 °C. After 24 h, the culture medium was removed and renewed with another 100 μL fresh medium per well. Nanofiber specimens, as well as drug suspensions in triplicate, were added to the wells. After 72 h, a fixation step was completed by discarding the media and adding 150 μL of 10%TCA, and then by incubating the plates at 4 °C for 1 h. Thereafter, the TCA was discarded entirely, and 5 cycles of washing with distilled water were performed. At this moment, each well of the plates received an aliquot of 70 μL SRB solution (0.4%*w*/*v*), and the plates were incubated in the dark at 25 °C for 10 min. Lastly, plates were washed thrice with 1% acetic acid and left to air-dry overnight. On the following day, 150 μL of TRIS (10 mM) was added per well in an attempt to dissolve the stain, and absorbance was read at 540 nm using a BMGLABTECH^®^-FLUOstar Omega microplate reader (BMG Labtech, Ortenberg, Germany). The concentration inhibiting 50% cell proliferation (IC_50_ value for cancer cells or CC_50_ value for normal cells) was computed using Sigma Plot 12.0 software (version 12.0 software (Systat Software Inc., San Jose, CA, USA).

### 2.10. Apoptosis Assay

Apoptosis Assay was executed by the utilization of an Annexin V-FITC apoptosis detection kit (Abcam Inc., Cambridge Science Park, Cambridge, UK) and a two fluorescent channels flow cytometry (ACEA Biosciences Inc., San Diego, CA, USA) as per the manufacturer’s guidelines. The test specimens were applied to the cells for 48 h only, and then they (10^5^ cells) were collected from each specimen and washed with ice-cold PBS (pH 7.4). As provided, 0.5 mL of Annexin V-FITC/PI was added to the cells, which were then left with the stain for 30 min in a dark place at 25 °C. Then, cells were injected into the flow cytometer to test for Annexin V-FITC/PI staining. The first signal detector FL1 was used to detect FITC, while the second signal detector FL2 was used for PI detection. A number of 12,000 events were acquired per sample, and quadrant analysis was computed using built-in software (ACEA NovoExpress™ software version 1.3.0, Biosciences Inc., San Diego, CA, USA)

### 2.11. Cell Cycle Analysis

The cell cycle analysis was performed as described in our previous work [33]. In brief, the test agents were applied to the cells for 48 h, and then an estimate of 10^5^ cells were collected per sample and washed with ice-cold PBS (pH 7.4). Then, 60% ice-cold ethanol was added to the cells and left for 1 h in the fridge for fixation. Thereafter, ethanol was removed, and cells were subjected to 2 cycles of washing with PBS (pH 7.4). The cells were then resuspended in 1 mL PBS, 50 μg/mL RNAase A, and 10 μg/mL propidium iodide (PI) solution and left in a dark incubator at 37 °C for 20 min. Eventually, cells were injected into the flow cytometer, an acquisition of 12,000 events per sample was applied, and cells were detected for the PI stain using FL2 (λex/em 535/617 nm) signal detector. Cell cycle distribution was computed using built-in software (ACEA Biosciences Inc., San Diego, CA, USA).

### 2.12. Statistical Analysis

GraphPad Prism 6 was used to perform all the required analysis. All experiments were performed in triplicates. The results were presented as the mean of these triplicates ± standard deviation (SD). ANOVA with multiple comparisons post hoc test was used to test the significance among normally distributed data. Non-parametrical Kruscal–Wallis and Mann–Whitney tests were used whenever the data deviated from the normal distribution. Statistical significance was considered at a *p*-value of ≤0.05. 

## 3. Results

### 3.1. Size and Morphology Characterization of Nanofibers

**Figure 1:**
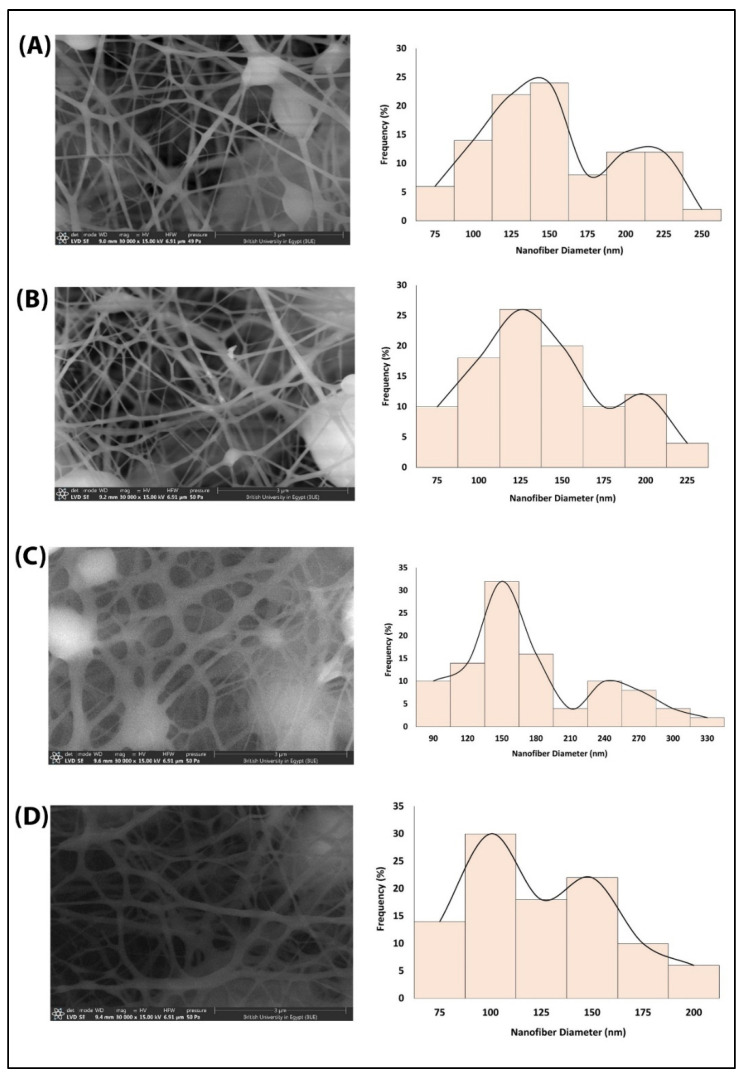
Scanning Electron Microscopy (SEM) images of the (**A**) PCL, (**B**) N@PCL, (**C**) L@PCL, and (**D**) N + L@PCL nanofibers with their diameter histograms produced utilizing the image processing program ImageJ (NIH, Bethesda, MD, USA).

The designed nanofiber mats underwent an SEM analysis to evaluate the morphological features and diameters of the free PCL, N@PCL, L@PCL, and N + L@PCL. As depicted in Figure 1, all fabricated nanofibers were randomly aligned, interconnected, and exhibited smooth surfaces. In addition, they showed homogeneity and uniformity of the ingredients, evident by the absence of any visible drug (N or L). This indicated the entire integration of either N, L, or N + L into the nanofibers’ matrices. The mean nanofiber diameters were studied using ImageJ’s image processing program (NIH, Bethesda, MD, USA). The fiber diameters in all the nanofiber films were in the same range of 63.4–231.6 nm, except the L@PCL nanofiber mat, which showed a wider diameter range of 67.3–300.9 nm. This may be explained by the reduced conductivity of the electrospun solution by adding L. Moreover, a bimodal pattern was observed in all nanofiber diameter histograms, with the thinner nanofibers showing higher peaks. The exact mean nanofiber diameter and diameter range of the fabricated nanofibers are presented in Table 1. Our findings are in accordance with previous studies [34,35].

### 3.2. Characterization and Loading Efficiency

**Figure 2:**
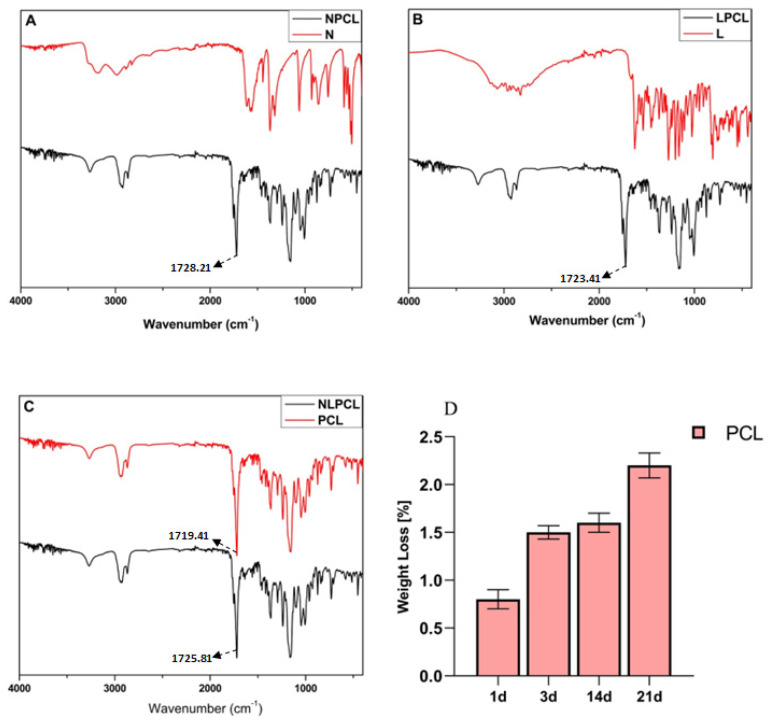
(**A**) FTIR spectra of (**A**) N and N@PCL, (**B**) L and L@PCL, and (**C**) PCL and N + L@PCL. (**D**) Weight loss percentages of the free PCL nanofiber at 37 °C in a phosphate buffer (pH 7.4). Data correspond to means ± SD (*n* = 3).

FTIR analysis was performed to study the chemical features of PCL, N@PCL, L@PCL, and N + L@PCL nanofibers (Figure 2A–C). The FTIR spectrum of PCL exhibited a sharp peak at 1719.41 cm^−1^ corresponding to the ester carbonyl stretching vibration and two peaks at 1238.58 and 1047.1 cm^−1^ relative to -C-O- ester. The FTIR spectrum of free L exhibited three peaks at 3100 (-NH), 2963.4 cm^–1^ (-CH stretching), and 1625.8 cm^–1^ (C=C stretching) [27,36]. The FTIR spectrum of N showed peaks at 3178.7 cm ^−1^ (-OH stretching), 2986 (CH stretching), 1756 cm^−1^ (C=O bond), 1367.4 cm^−1^, and 1059.7 cm^−1^ (C=C) [37,38,39]. Since the amount of N and L was much lower than that of PCL in the nanofibers, the major peaks of N and L were masked by the vibrations of the PCL substrate [35]. Based on the structure of the N and active alkaloids of L, all the chemical structures contain H-bond donating functional groups capable of interacting with the ketonic group of the PCL. Such interaction is hypothesized to enhance the physical loading of the drugs. The spectra of the N and/or L-loaded nanofibers showed a shifting of the ester carbonyl stretching vibration peak compared to that of the free PCL nanofibers. After N-loading, L-loading, and N/L-loading, the peak position shifted from 1719.41 cm^−1^ to 1728.21, 1723.41, and 1725.81 cm^−1^, respectively, which agrees very well with previously reported studies [35,38,39].

**Figure 3:**
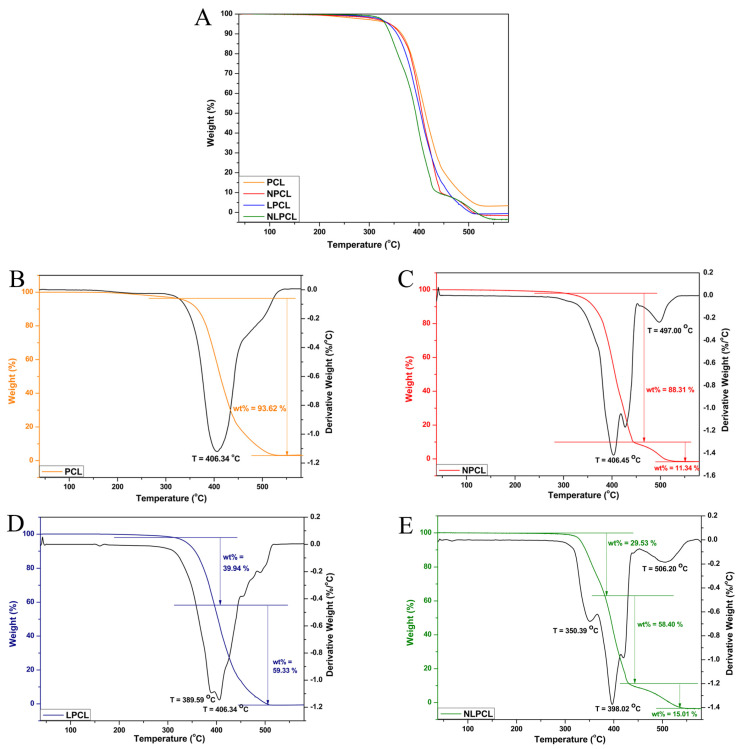
(**A**) Thermogravimetric analysis (TGA) spectra of the free PCL, N@PCL, L@PCL, and N + L@PCL nanofibers. TGA-DTG profiles of the (**B**) free PCL, (**C**) N@PCL, (**D**) L@PCL, and (**E**) N + L@PCL nanofibers.

TGA was performed to investigate the thermal behavior of the free PCL, N@PCL, L@PCL, and N + L@PCL nanofibers, revealing the mass loss of each fiber sample with increasing temperature. The weight loss versus temperature increment is presented in Figure 3A. All nanofiber samples displayed high stability at elevated temperatures beyond 300 °C. At 100 °C, no weight loss was observed, indicating that the nanofiber samples were free of trapped moisture, possibly due to the PCL polymer’s hydrophobic nature. The derivative thermogravimetric (DTG) analyses of the produced TGA curves were plotted, and the TGA-DTG curves are shown in Figure 3B–E. It can be observed that the free PCL nanofibers show only one peak detected in the plot, characterizing the thermal destruction of PCL with 93.62% weight loss at 406.34 °C (Figure 3B). Two peaks were presented in the N@PCL and L@PCL plots, as displayed in Figure 3C and 3D, respectively. In both cases, one of the two peaks showed the thermal destruction of PCL nanofibers at 406.45 °C and 406.34 °C in N@PCL and L@PCL plots, respectively. In the N@PCL plot, 11.34% weight loss was observed at 497.00 °C, attributed to the destruction of N. In the L@PCL plot, 39.94% weight loss was observed at 389.59 °C, attributed to the destruction of harmala extract.

On the other hand, the N + L@PCL plot presents a three-stage sample thermal destruction (Figure 3E) of the harmala extract, PCL, and Nedaplatin at 350.39, 398.02, and 506.20 °C, respectively. The thermal vulnerability shift of the dual-loaded PCL nanofibers is trivial, indicating their physical stability with the loaded compounds. These results are in line with previous similar studies [13,40,41]. 

The loading efficiency values (%) of N in N@PCL, N + L@PCL, and L in L@PCL and N + L@PCL were found to be 99.4, 99.8, 99.6, and 99.9%, respectively. These findings agree with previous work demonstrating that almost 100% of the drugs are either loaded or adsorbed onto PCL nanofibers [42]. 

### 3.3. In Vitro Degradation Study

The in vitro degradation study of electrospun PCL nanofibers was performed to study the PCL degradation behavior in PBS solution (pH 7.4) for 21 days. Figure 2D demonstrates the weight loss percentage of the free PCL, calculated from the difference between the initial sample weight and the weights at each time point (1 day, 3 days, 14 days, and 21 days). The presented data correspond to means ± SD of the experimental triplicate. The PCL nanofibers’ degradation reflects the hydrolytic degradation of polymeric chains resulting from the hydrolysis of ester bonds. Since generally, the degradation rate of a biopolymer scaffold varies with a range of factors, including molecular weight, crystallinity, morphology, and hydrophilicity [43]. These results align very well with previously reported studies concerning PCL nanofibers’ degradation behavior [35,44]. 

### 3.4. Release Study

**Figure 4:**
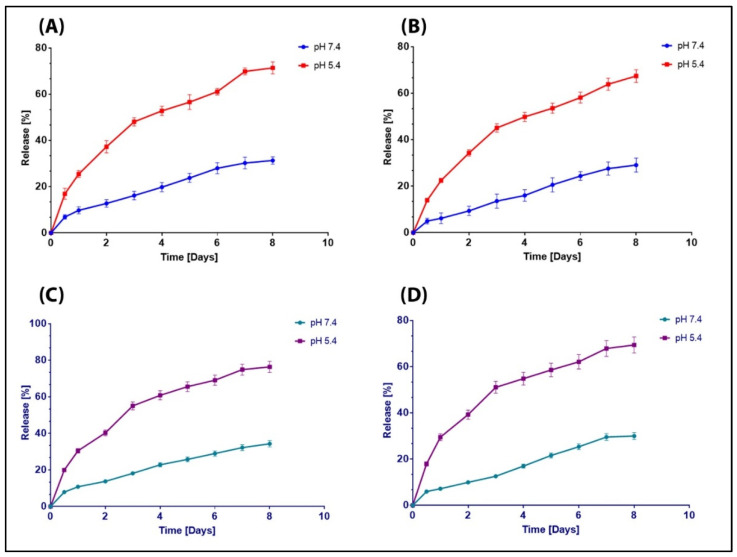
Release [%] of (**A**) N from N@PCL, (**B**) L from L@PCL, (**C**) N from N + L@PCL and (**D**) L from N + L@PCL at 37 °C into a phosphate buffer (pH 7.4) and acetate buffer (pH 5.4). Data correspond to means ± SD (*n* = 3).

Figure 4 presents N and L release profiles from N@PCL, L@PCL, or N + L@PCL nanofiber mats into a phosphate buffer (pH 7.4) and acetate buffer (pH 5.4) at 37 °C. All the release data correspond to means ± SD (*n* = 3). At pH 5.4, the release of both drugs from either electrospun nanofiber mats revealed a biphasic release pattern for 8 days. In the first 3 days, rapid diffusion of N occurred from N@PCL (48.05 ± 1.81%) and from N + L@PCL (55.05 ± 2.20%). This rapid release was followed by a sustained release of N from N@PCL (61.10 ± 1.40% after 6 days, and 71.37 ± 2.57%, after 8 days) and from N + L@PCL (69.10 ± 2.76%, after 6 days, and 76.37 ± 3.06%, after 8 days). Likewise, rapid diffusion of L occurred from L@PCL (45.05 ± 1.80%) and from N + L@PCL (51.05 ± 2.55%). Also, this rapid release was followed by a sustained release of L from L@PCL (58.10 ± 2.32%, and 67.37 ± 2.70%, after 6 and 8 days, respectively) and from N + L@PCL (62.10 ± 3.11%, and 69.37 ± 3.47%, after 6 and 8 days, respectively). The initial rapid release of the loaded drugs may be due to their location in the outer surface as well as in the near-inner surface of the nanofibers. In addition, the high porosity of the nanofiber mats enhances the flow of the dissolution medium between the nanofiber networks. Also, the small diameter of the nanofibers results in a large surface area-to-volume ratio, increasing the interaction points of the loaded drugs with the dissolution medium. The following sustained release of the drugs is attributed to their entrapment in the center of the nanofibers, which are then slowly released due to PCL degradation by erosion and diffusion. These results are in accordance with previously published studies of the release rate of a drug from PCL nanofibers [34,45]. 

The degradation of PCL, like many biodegradable polymers, occurs via ester bond hydrolysis with the production of carboxyl and hydroxyl groups in nanofibers. The production of basic groups explains the faster release rate in acetate buffer, at pH 5.4, compared to the release rate in PBS, at pH 7.4. The basic groups may be consumed by the protons in the acidic medium, which shifts the degradation reaction forward, thus increasing the erosion of the nanofibers in acetate buffer and resulting in a quicker drug release [46]. The higher and faster release rates of the payloads in acidic cancerous pH compared to that in physiological pH suggests the selective release manner in cancer cells rather than in normal cells, which reduces the undesired effects [47]. In addition, the locally sustained release behavior of N and L from the electrospun PCL mats can keep a local therapeutic concentration within cancer cells while minimizing off-target effects [48].

### 3.5. Release Kinetics

**Figure 5:**
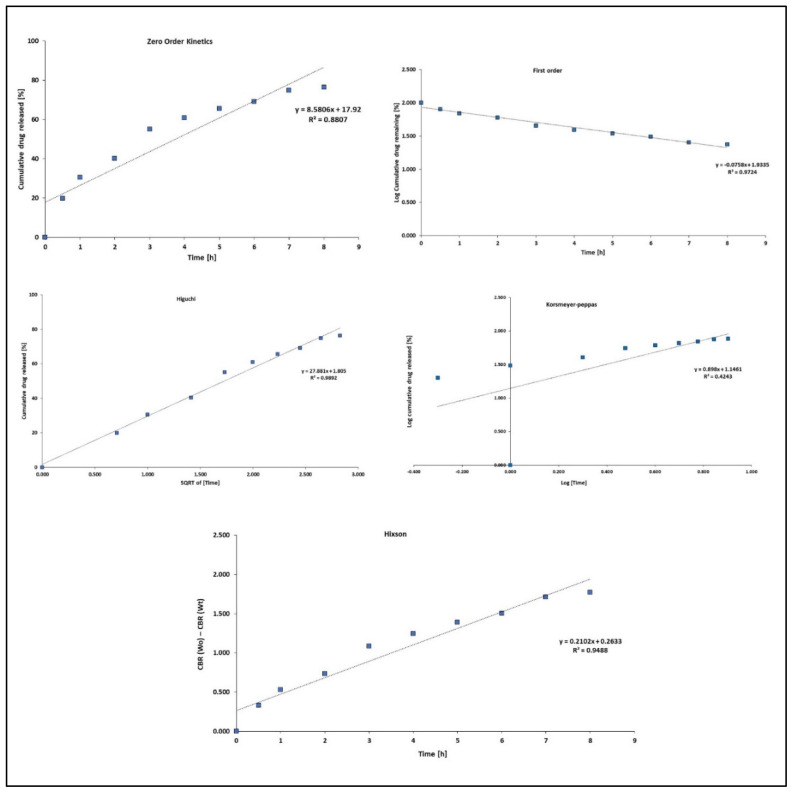
Release kinetics of N from N + L@PCL tailored to five kinetic models.

**Figure 6:**
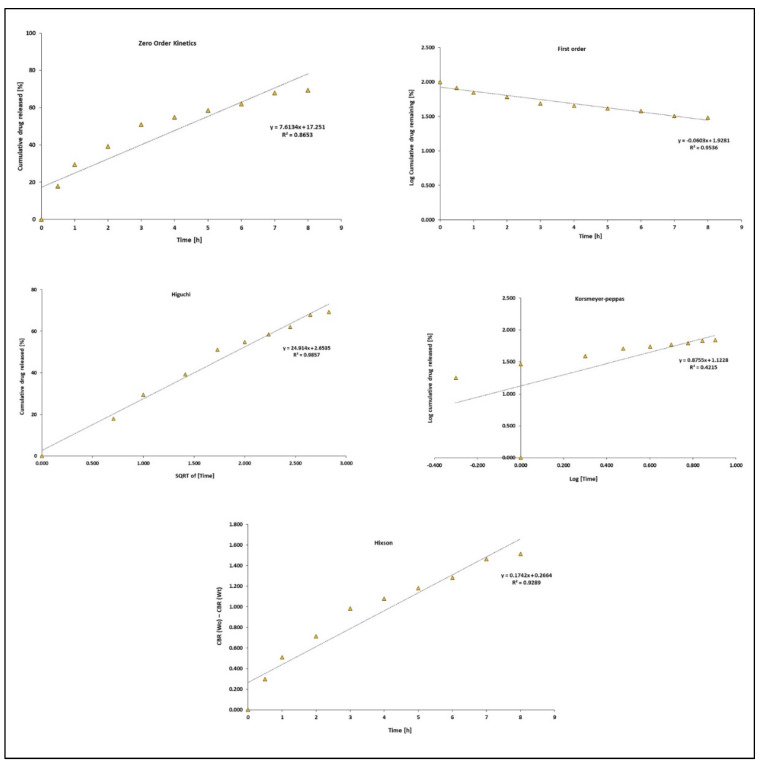
Release kinetics of L from N + L@PCL tailored to five kinetic models.

To further examine the release mechanism of N and L from N + L@PCL fabricated electrospun nanofiber mats in an acidic solution of pH 5.4, various mathematical kinetics models were applied, including the zero-order, first-order, Higuchi, and Hixson models (Figure 4 and Figure 5). Table 2 reveals the relevant kinetic release coefficients (R^2^ and *K*). In addition, the data acquired from the in vitro release study were fitted to the Korsmeyer–Peppas model to acquire more knowledge regarding the type of diffusion mechanism of the N and L from N + L@PCL nanofibers. The diffusional exponent (*n*) acquired from the Korsmeyer–Peppas model shed more light on the payload release mechanism [49], where *n* ≤ 0.45 indicates Fickian diffusion and 0.45 < *n* < 1 indicates a non-Fickian model, indicating irregular transport [50,51]. This complementary model gives clues on the drug release behavior from polymeric systems when more than one type of release manner is involved. In our study, and as depicted in Figure 5 and Figure 6 and Table 2, it was shown that the N and L release from N + L@PCL nanofibers, in acidic pH best followed the Higuchi diffusion equation with the highest R^2^ values among all kinetic models (R^2^ values of 0.99 and 0.98 in the case of N from N + L@PCL and L from N + L@PCL, respectively). These findings propose that the diffusion mechanism dominantly governed the release manner of both N and L from the electrospun nanofibers. Then, the data obtained from fittings into the Korsmeyer–Peppas model indicated a non-Fickian diffusion release mechanism as their *n* values were 0.89 and 0.87 in the case of N from N + L@PCL and L from N + L@PCL, respectively. This suggested the irregular diffusion of the payloads (N and L) from the electrospun PCL nanofibers. These findings showed that the drugs were released from the polymeric matrices via erosion and diffusion mechanisms since the irregular diffusion suggests a combination of both diffusion and erosion drug release manners [51]. These findings align with the in vitro release study and previously reported studies [31,52]. 

### 3.6. Cytotoxicity Assay

Nedaplatin is currently implemented in chemotherapy treatment protocols for several tumors, including head, neck, esophagus, and lung cancers [53]. Moreover, clinical trials postulated that N mended some of the advanced breast cancer patients’ sufferings through the extension of time-to-treatment failure (TTF), together with increasing the overall survival (OS) [17]. *P. harmala* has also reported significant antiproliferative activity against MCF-7 and MDA-MB-231 breast cancer cell lines [54]. 

Herein, the breast cancer MCF-7 cell line was treated by different drug-loaded nanofibers for 72 h to measure their cytotoxic activity. Moreover, the free drugs (L, N) were used as positive controls, while the normal cell line (MCF-10a) was used to assess the selectivity in the cytotoxic activity by the developed nanofibers. The findings demonstrated that the L@PCL nanofiber possessed a comparable antiproliferative activity to the free drug counterpart against MCF-7 cells. However, the N@PCL nanofiber exhibited better cytotoxic activity relative to the free drug. Moreover, the enhanced antiproliferative cytotoxicity against MCF-7 cells was also attained by the dual-loaded N + L@PCL nanofiber, as shown in Table 3 and Figures S1 and S2.

The selectivity index (SI) is a quantitative reflection of the extent to which the drug-loaded nanofibers can selectively affect the cancer cell lines while sparing the normal counterparts. This means that the higher the SI value, the better the selectivity. The results show that all the developed drug-loaded nanofibers exhibited high selectivity, where the highest SI was attained by the dual drug-loaded nanofiber (N + L@PCL) followed by L@PCL and then N@PCL nanofibers, respectively (Table 3). 

Collectively, it can be inferred that using the nanofiber loaded with the drug combination indeed resulted in an overall positive outcome. The results show that N + L@PCL exhibited an enhanced antiproliferative activity while reducing the dosages required to produce the same pharmacodynamic effect. Also, the significantly improved selectivity suggests a potential lowering in the treatment-induced adverse effects.

### 3.7. Apoptosis Assay

**Figure 7:**
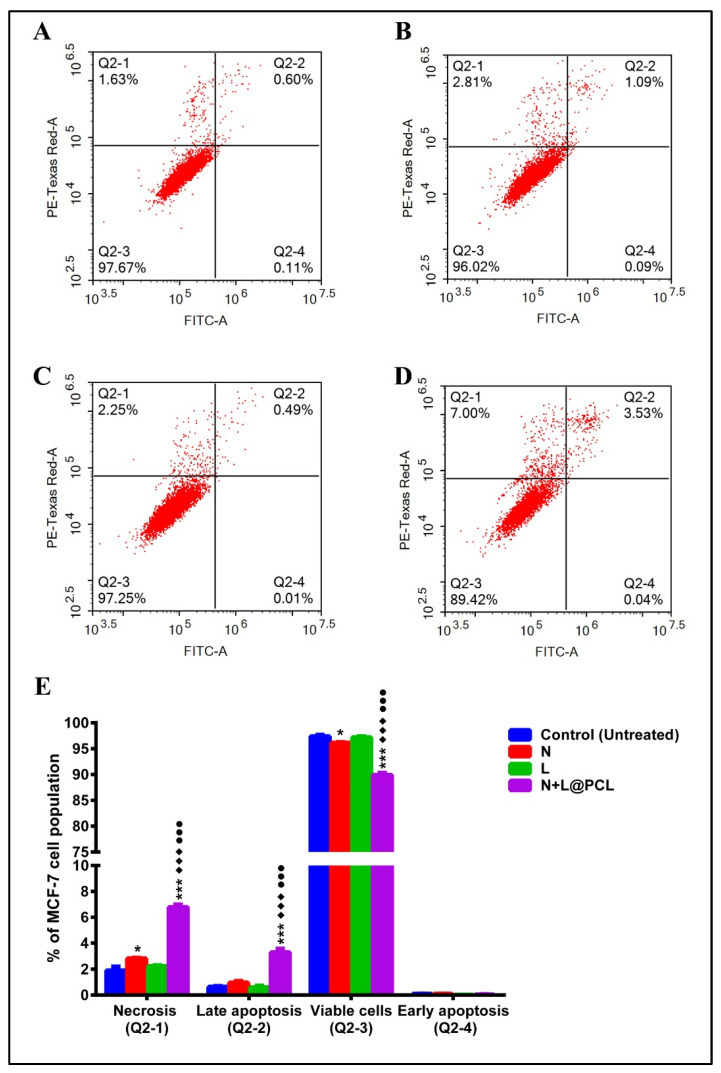
A display of the apoptosis assay results in MCF-7 cells when incubated for 48 h with the tested compounds (N, L, and N + L@PCL). Cytograms presenting annexin-V/Propidium Iodide-stained untreated MCF-7 cells as negative control (**A**), cells treated with N (**B**), cells treated with L (**C**), cells treated with N + L@PCL (**D**), and analysis of the four quartiles resulting from apoptosis assay (**E**). Quadrant charts show Q2-1 (necrotic cells, AV−/PI+); Q2-2 (late apoptotic cells, AV+/PI+); Q2-3 (normal cells, AV−/PI−); Q2-4 (early apoptotic cells, AV+/PI−). Data are the average of three individual experiments ± standard deviation (SD). Asterisks ***, * refer to significant differences from the control where *p*-values are ≤0.001, ≤0.01, and ≤0.05, respectively. Symbols ♦♦♦, refer to significant differences from N where *p*-values are ≤0.001, ≤0.01, and ≤0.05, respectively. Symbols ●●●, refer to significant differences from L where *p*-values are ≤0.001, ≤0.01, and ≤0.05, respectively.

Guided by the cytotoxicity results, the optimum drug-loaded nanofiber (N + L@PCL) was tested for the induction of apoptotic cell death in MCF-7 cell. For this purpose, untreated cells were used as a negative control (Figure 7A), whereas the free drugs (N, L) were used as positive controls (Figure 7B,C), as compared to N+L@PCL (Figure 7D). Forty-eight hours post-treatment, cells were labeled by annexin-V and PI and analyzed by flow cytometry. The results confirmed that N + L@PCL significantly reduced the overall cell viability (Q2-3) compared to all other control groups (*p*-value of ≤0.001 in all cases) (Table 4 and Figure 7E). N + L@PCL induced cell death through apoptosis (Figure 7D). Hence, the double-stained cell population undergoing late apoptosis (Q2-2) is significantly higher in the case of N + L@PCL treated cells (*p*-value of ≤0.001 in all cases) compared to all other treatment groups. The obtained results again emphasize the advantage of using a drug combination and affirm the previously reported capability of both drugs (N, L) to induce apoptosis [15,24]. The apoptotic effects of N on gynecologic cancer cells such as ovarian (Skov-3) and cervical (Hela), as well as esophageal cancer (Eca-109) have been anticipated before [55]. A former study by Shabani and colleagues reinforced the ability of L to promote apoptosis in the triple-negative invasive MDA-MB-231 breast cancer cell line by modulating both intrinsic and extrinsic pathways of apoptosis [25]. 

In this study, N + L@PCL stimulated cell death through an additional necrotic mechanism where the PI-labelled cells (Q2-1) were 3–3.5 folds higher as compared to the other groups (*p*-value of ≤0.001 in all cases). Importantly, compared to the untreated control, the N-treated cells exhibited reduced cell viability (*p*-value of ≤0.05), which is mainly mediated through necrosis (*p*-value of ≤0.05) (Table 4 and Figure 7E).

### 3.8. Cell Cycle Analysis

**Figure 8:**
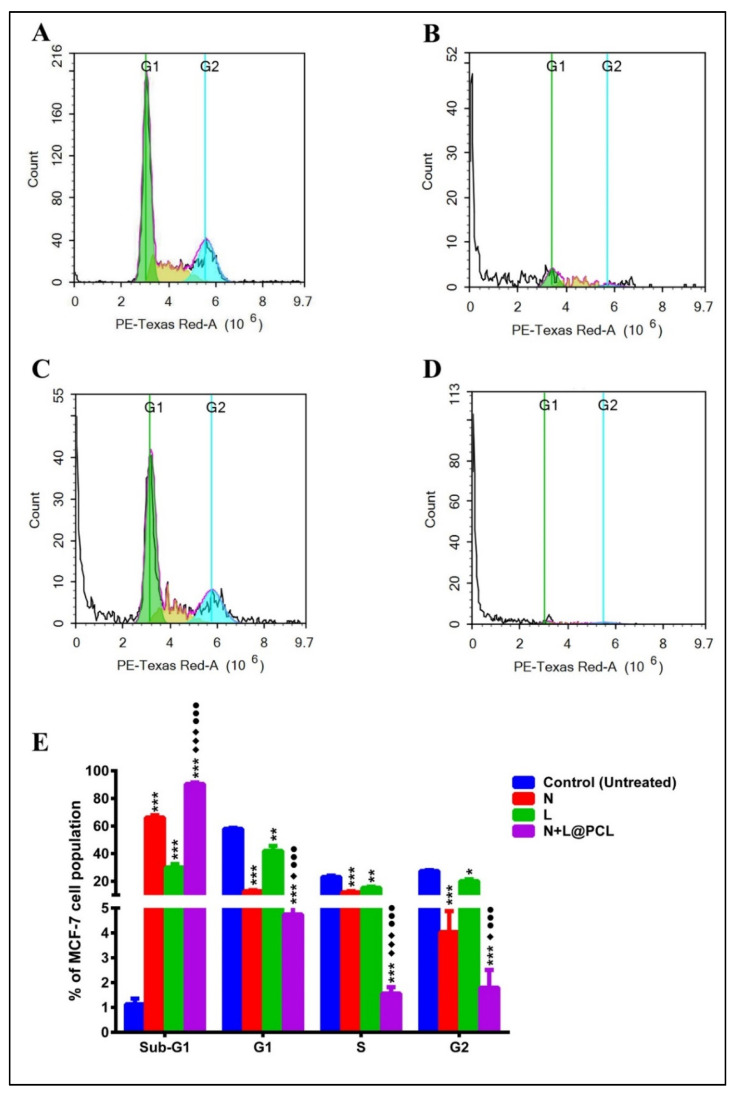
Cytogram showing cell cycle analysis of MCF-7 cells after 48 h incubation of nontreated MCF-7 cells (control) (**A**), MCF-7 cells treated with N (**B**), MCF-7 cells treated with L (**C**), MCF-7 cells treated with N + L@PCL (**D**), and percentage of MCF-7 cell population in various cell cycle phases (**E**). Data are the average of three individual experiments ± standard deviation (SD). ***, **, * refer to significant differences from the control where *p*-values are ≤0.001, ≤0.01, and ≤0.05, respectively. Symbols ♦♦♦, ♦ refer to significant differences from N where *p*-values are ≤0.001, ≤0.01, and ≤0.05, respectively. Symbols ●●●, refer to significant differences from L where *p*-values are ≤0.001, ≤0.01, and ≤0.05, respectively.

Cell cycle analysis was also examined to better understand the mechanism by which N + L@PCL promotes cytotoxicity against MCF-7 cells (Figure 8A–D). The followed treatment protocol, as well as the controls used, were similar to the apoptosis assay. Based on the obtained data, it was found that N + L@PCL nanofiber significantly induced cell cycle arrest at the sub-G1 phase (*p*-value of ≤0.001 in all cases) (Table 5 and Figure 8E). Surprisingly, both of the individual free drugs (N, L) abrogated the cell cycle at the same phase (sub-G1), suggesting a similar mechanism of cytotoxicity yet to a lesser extent relative to the dual drug-loaded electrospun fiber (*p*-value of ≤0.001, relative to the untreated control for both drugs) (Figure 8E). The observed halting of cell cycle progression at the sub-G1 phase suggests that both drugs can bind to and damage the DNA, resulting in apoptosis [56]. Similar findings were reported for harmaline, which excelled at halting the cell cycle in sub-G1 and promoting apoptosis in U-87 glioblastoma cells [57]. In addition, N treatment resulted in a remarkable increase in the non-small cell lung cancer population in the G_0_/G_1_ phase, whereby they interfered with the first stage of the cell cycle [58]. On the contrary, applying N treatment to AZ-521 human gastric cancer cell lines resulted in a reduction in the G_0_/G_1_ population with no profound effect on the G_2_/M population [59]. The researchers described their findings in terms of the ability of N to oppose S to G2/M and G2/M to G1 transitions in gastric cancer cells [59]. 

In a nutshell, the free drugs (N, L), as well as the dual drug-loaded electrospun fiber (N + L@PCL), blocked the MCF-7 cell cycle progression by accumulating the cells in the sub-G1 phase, and the effects were shown to be maximized with the N + L@PCL nanofiber. The findings were in agreement with recent studies involving L and N on glioblastoma and non-small cell lung cancer, respectively [57,58]. All of which confirm our reported results of the apoptosis induction assay. 

## 4. Conclusions

Using phytochemicals in adjuvant to chemotherapeutics could be a promising approach to boost the therapeutic effect against breast cancer while preventing its recurrence. Moreover, this tactic would minimize administering high doses of chemotherapeutics and thus reduce their potential toxic effects. Here, N + L-loaded PCL-based electrospun nanofibers were fabricated for the pH-dependent sustained release of payloads in cancer cells. The designed nanofibers exhibited nano-diameters, low degradation under physiological conditions, and outstanding thermal stability. In addition, N + L@PCL demonstrated the highest cytotoxic and apoptotic effects on MCF-7 breast cancer cells compared to free drugs and PCL-based electrospun nanofibers loaded with single therapy. The cell cycle kinetics provided further evidence for the apoptosis and cytotoxicity assays where the dual-loaded PCL-based electrospun nanofibers trapped MCF-7 cells at the sub-G1 phase. As such, this study suggests the potential use of the designed nanofiber mats as perfect implants to prevent tumor recurrence after surgical removal of breast tumors.

## Figures and Tables

**Table 1:** Mean nanofiber diameter and diameter range of the free PCL, N@PCL, L@PCL, and N + L@PCL nanofibers.

Nanofiber Film	Mean Nanofiber Diameter (nm) ± SD	Nanofiber Diameter Range (nm)
PCL	140.4 ± 44.9	64.7–231.6
N@PCL	128.1 ± 40.8	63.4–204.2
L@PCL	157.9 ± 60.7	67.3–300.9
N + L@PCL	112.0 ± 34.5	64.3–184.3

**Table 2:** Rate constants and correlation coefficients (*R*^2^) of N and L released from N + L@PCL were calculated by fitting into various release models (zero-order, first-order, Higuchi, Korsmeyer–Peppas, and Hixson models).

Models	N from N + L@PCL	L from N + L@PCL
**Zero-order**		
*k*_0_	8.58	7.61
*R* ^2^	0.88	0.86
**First-order**		
*k_f_*	0.08	0.06
*R* ^2^	0.97	0.95
**Higuchi**		
*k_H_*	27.88	24.91
*R* ^2^	0.99	0.98
**Korsmeyer–Peppas**		
n	0.89	0.87
*R* ^2^	0.42	0.42
**Hixson**		
κ t	0.21	0.17
*R* ^2^	0.95	0.93

**Table 3:** Cytotoxicity against MCF-7 and MCF-10A cell lines after incubation with the test specimens for 72 h. Data are presented as the average of triplicates ± standard deviation (SD).

Test Sample	IC_50_ on MCF-7 Cells (µg/mL)	CC_50_ on MCF10A Cells (µg/mL)	SI (CC_50_/IC_50_)
L	4.93 ± 0.13	62.53 ± 1.38	12.68
N	4.59 ± 0.37	24.24 ± 1.15	5.28
L@PCL	4.40 ± 0.56	67.07 ± 1.64	15.24
N@PCL	3.42 ± 0.17	27.92 ± 0.92	8.16
N + L@PCL	3.21 ± 0.86	65.63 ± 1.29	20.45

**Table 4:** Apoptotic assay results for MCF-7 cells after 48 h exposure to the test compounds.

Apoptotic Stage	Control (Untreated)	N	L	N + L@PCL
Necrosis (Q2-1)	1.903 ± 0.3035	* 2.830 ± 0.04359	2.223 ±0.08327	***♦♦♦●●● 6.773 ± 0.1986
Late apoptosis (Q2-2)	0.6367 ± 0.05508	0.9600 ± 0.1253	0.5833 ±0.1286	***♦♦♦●●● 3.300 ± 0.2524
Viable cells (Q2-3)	97.37 ± 0.2950	* 96.12 ± 0.1168	97.18 ±0.1909	***♦♦♦●●● 89.88 ± 0.4148
Early apoptosis (Q2-4)	0.0900 ± 0.01732	0.0900 ± 0.0200	0.01333 ±0.005774	0.0400 ± 0.0200

The provided percent is the mean of triplicate independent runs ± standard deviation. A *p*-value of ≤0.05 is considered statistically significant. The symbols (*), (♦), and (●) refer to statistical significance from the Control, N, and L, respectively. Repetition of any symbol once only refers to a *p*-value of ≤0.05, twice only refers to a *p*-value of ≤0.01, and thrice refers to a *p*-value of ≤0.001, respectively.

**Table 5:** Cell cycle phases of MCF-7 cells after exposure to N, L, and N + L@PCL for 48 h.

Phases	Control (Untreated)	N	L	N + L@PCL
Freq Sub-G1	1.127 ± 0.2309	*** 66.09 ± 1.752	*** 29.99 ± 2.435	***♦♦♦●●● 90.44 ± 1.172
Freq G1	57.89 ± 0.7967	*** 12.81 ± 0.659	** 42.03 ± 3.516	***♦●●● 4.740 ± 2.941
Freq S	23.16 ± 0.9295	*** 11.92 ± 0.8504	** 15.28 ± 0.8675	***♦♦♦●●● 1.557 ± 0.260
Freq G2	27.45 ± 0.6293	*** 4.033 ± 0.842	* 20.11 ± 1.364	***♦●●● 1.800 ± 0.7118

The displayed data are the mean of triplicate individual runs ± standard deviation (SD). Freq stands for frequency of cells at the different phases of cell cycle. The symbols (*), (♦), and (●) refer to statistical significance from the Control, N, and L, respectively. Repetition of any symbol once only refers to a *p*-value of ≤0.05, twice only refers to a *p*-value of ≤0.01, and thrice refers to a *p*-value of ≤0.001, respectively.

## Data Availability

Data is contained within this article.

## Supplementary Materials

The following supporting information can be downloaded at: https://www.mdpi.com/article/10.3390/pharmaceutics15102367/s1. Figure S1. UV Spectra of (A) L and (B) N; Figure S2. Dose-response curves show the % viability of MCF-7 after exposure to the test specimens; Figure S3. Dose-response curves show the % viability of normal breast epithelial cell lines (MCF10A) after exposure to the test specimens.
