# Supplementary material for: Nedaplatin/Peganum harmala Alkaloids Co-Loaded Electrospun, Implantable Nanofibers: A Chemopreventive Nano-Delivery System for Treating and Preventing Breast Cancer Recurrence after Tumorectomy
Source: Pharmaceutics. 2023 Sep 22;15(10):2367. doi: 10.3390/pharmaceutics15102367 (PMC10609766; doi:10.3390/pharmaceutics15102367)
Supplement: Supplementary file 1 [file pharmaceutics-15-02367-s001.zip › pharmaceutics-2564063-supplementary.pdf]

Supplementary Materials

Nedaplatin/*Peganum harmala* Alkaloids Co-Loaded Electrospun, Implantable Nanofibers: A Chemopreventive Nano-Delivery System for Treating and Preventing Breast Cancer Recurrence after Tumorectomy

Nada K. Sedky [1,†], Kholoud K. Arafa [2,†], Manal M. M. Abdelhady [3], Marwa Y. Issa [4], Nour M. Abdel-Kader [1,5], Noha Khalil Mahdy [6], Fatma A. Mokhtar [7], Mohammad Y. Alfaifi [8] and Sherif Ashraf Fahmy [9,*]

1 Department of Biochemistry, School of Life and Medical Sciences, University of Hertfordshire Hosted by Global Academic Foundation, R5 New Garden City, New Administrative Capital, Cairo 11835, Egypt

2 Drug Design and Discovery Lab, Zewail City for Science, Technology and Innovation, Cairo 12578, Egypt

3 Clinical Pharmacy Department, Faculty of Pharmacy, Badr University, Cairo 11829, Egypt

4 Department of Pharmacognosy, Faculty of Pharmacy, Cairo University, Kasr El-Aini Street, Cairo 11562, Egypt

5 Department of Biochemistry, Faculty of Science, Ain Shams University, Cairo 11566, Egypt

6 Department of Pharmaceutics and Industrial Pharmacy, Faculty of Pharmacy, Cairo University, Kasr El-Aini Street, Cairo 11562, Egypt

7 Department of Pharmacognosy, Faculty of Pharmacy, El Saleheya El Gadida University, El Saleheya El Gadida 44813, Egypt

8 Biology Department, Faculty of Science, King Khalid University, Abha 9004, Saudi Arabia

9 Department of Chemistry, School of Life and Medical Sciences, University of Hertfordshire Hosted by Global Academic Foundation, R5 New Garden City, New Capital, Cairo 11835, Egypt

* Correspondence: sheriffahmy@aucegypt.edu; Tel.: +20-1222613344

† These authors contributed equally to this work.

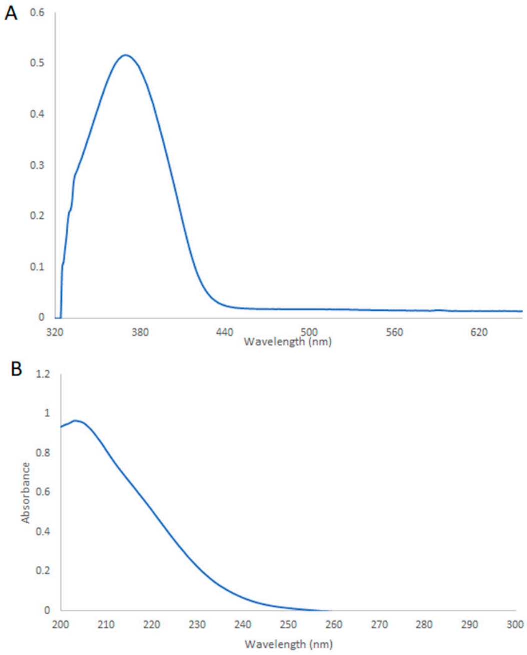

Figure S1. UV Spectra of (A) L and (B) N

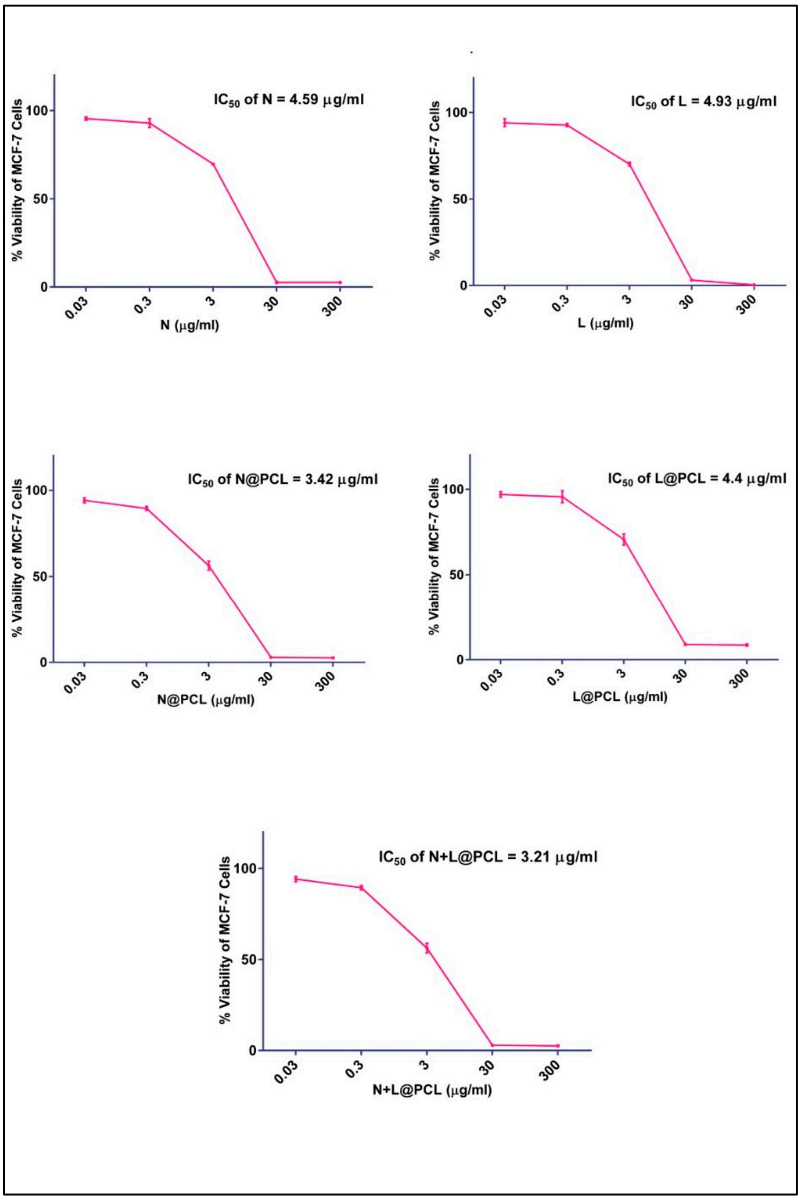

Figure S2. Dose-response curves show the % viability of MCF-7 after exposure to the test specimens.

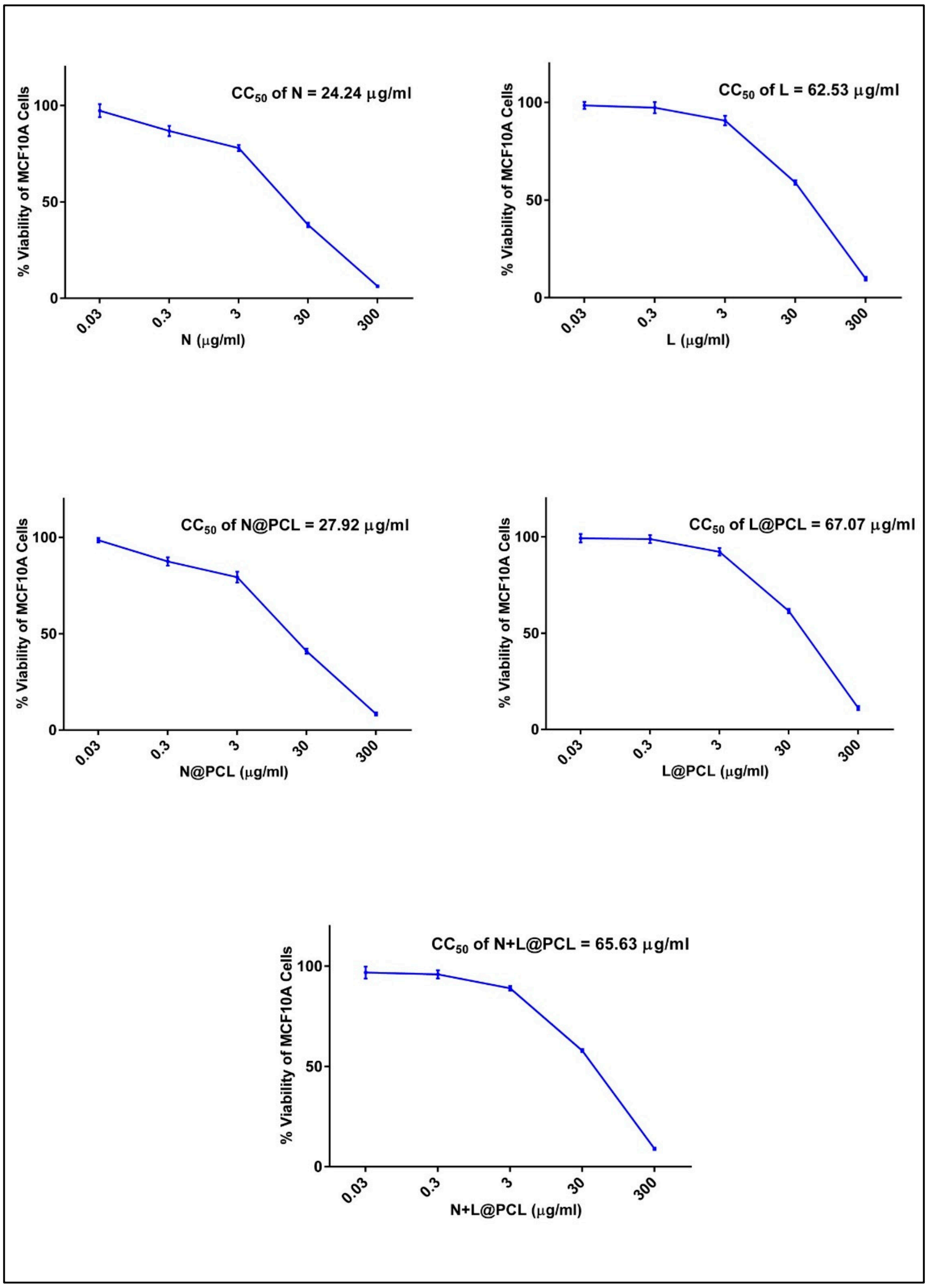

Figure S3. Dose-response curves show the % viability of normal breast epithelial cell lines (MCF10A) after exposure to the test specimens.
